# Refractory peptic ulceration following radiation therapy in primary gastric lymphoma: A report of two cases

**DOI:** 10.3892/ol.2014.2657

**Published:** 2014-11-03

**Authors:** CHUNYAN ZENG, SHIWEN LUO, NONGHUA LV, YOUXIANG CHEN

**Affiliations:** 1 Department of Gastroenterology, First Affiliated Hospital of Nanchang University, Nanchang, Jiangxi 330006, P.R. China; 2 Center for Experimental Medicine, First Affiliated Hospital of Nanchang University, Nanchang, Jiangxi 330006, P.R. China

**Keywords:** gastric lymphoma, radiotherapy, refractory peptic ulceration

## Abstract

The optimal prognosis for primary gastric lymphoma (PGL) is observed in those patients exhibiting PGL with minimal infiltration and who are eligible for radical resection. The initial treatment strategy for high-grade PGL (stages I/II) is chemotherapy followed by radiotherapy, however, subsequent to chemotherapy and/or radiotherapy for PGL, there is a risk of gastric bleeding and perforation. The present study reports two cases of PGL with refractory peptic ulcers that were negative for *Helicobacter pylori* following radiotherapy. Although the two patients received regular treatment for their ulcers and symptoms, the position and size of the ulcers remained unchanged for a number of years.

## Introduction

Malignant lymphomas include Hodgkin’s and non-Hodgkin’s lymphomas, which affect the stomach as either primary cancers or as part of a more widespread disease process. The stomach is also the most common site of secondary lymphoma (1,2). Compared with other gastric cancers, primary gastric lymphoma (PGL) is increasingly common and accounts for 2–8% of malignant gastric carcinomas (3–5). Currently, chemotherapy followed by radiotherapy is the preferred initial treatment strategy for high-grade stage I/II PGL, as opposed to surgery, and chemotherapy alone is administered for advanced-stage PGL cases (6,7). The present study describes two cases of PGL with intractable peptic ulceration following radiotherapy.

## Case report

### Case one

**Figure 1:**
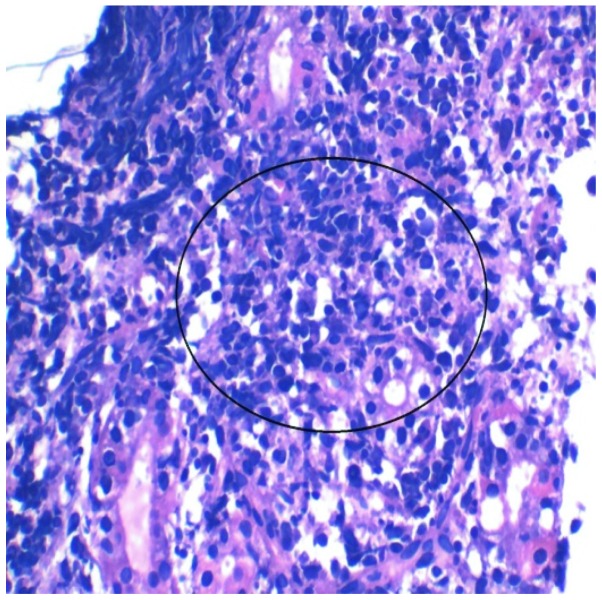
Case one: Hematoxylin and eosin staining of a gastric lesion revealed a primary malignant gastric lymphoma confined to the mucosa (circled) (magnification, ×400).

A 73-year-old female was admitted to the First Affiliated Hospital of Nanchang University (Nanchang, China) with a one-month history of upper abdominal distension and discomfort on September 5, 2003. In addition, the patient had a history of diabetes mellitus and hypertension. A computed tomography (CT) scan demonstrated extensive thickening of the mucosa and a mass on the posterior wall of the stomach that was indicative of gastric lymphoma. Gastroduodenoscopy (GIF-2T240; Olympus Corporation, Tokyo, Japan) and biopsy identified the mass to be a malignant gastric lymphoma (Fig. 1), and the initial laboratory analyses revealed the following data (normal ranges are provided in parentheses): Cancer antigen 125, 109 kIU/l (0–35 kIU/l); white blood cell count, 3.7×10^9^/l (4–10×10^9^/l); red blood cell count (RBC), 3.06×10^12^/l (3.5–6×10^12^/l); and lymphocyte percentage, 15.4% (18–58.4%). The patient received six courses of chemotherapy as follows: 375 mg/m^2^ rituximab on the first day; then a CHOP chemotherapy regimen consisting of 750 mg/m^2^ cyclophosphamide (CTX), 1.5 mg/m^2^ vincristine (VCR), 60 mg/m^2^ Pharmorubicin on day 1 and 30 mg prednisone on days 1–5, in total each course of chemotherapy lasted for three weeks, for a total of six courses. This was then followed by daily radiotherapy at 1.8 Gy for 20 days.

**Figure 2:**
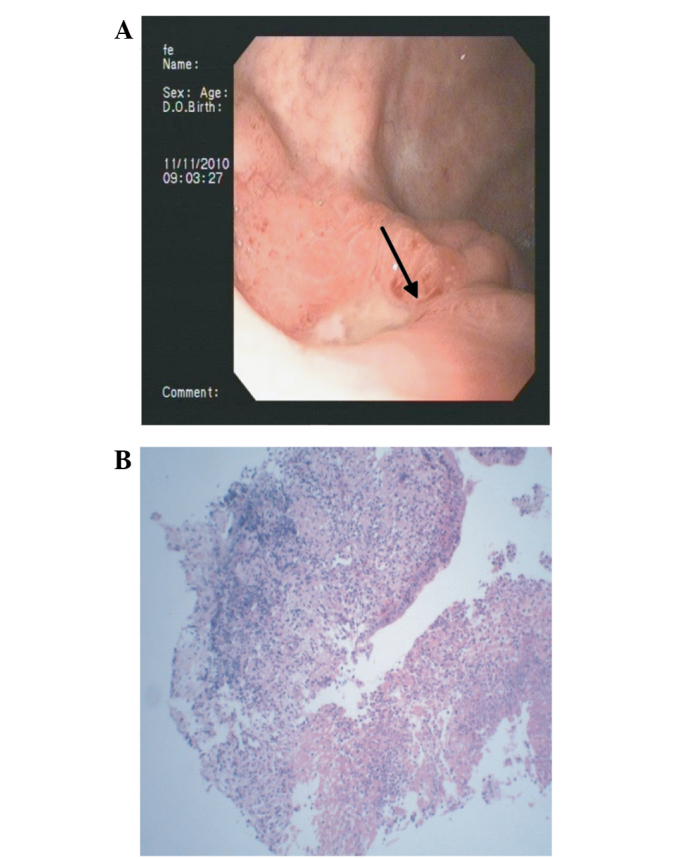
Case one: (A) Gastroduodenoscopy demonstrating an ulcer on the fundus of the stomach (arrow) and (B) hematoxylin and eosin staining of the lesion revealing it to be an ulcer accompanied by inflammation (magnification, ×100).

The patient was admitted to the hospital for a second time on November 25, 2010. The patient received a gastroscopical examination and was identified to be positive for *Helicobacter pylori* using the rapid urease test method, indicating that an ulcer was present on the fundus of the stomach (Fig. 2A); the ulcer was confirmed by performing a biopsy (Fig. 2B). Furthermore, a CT scan revealed a small hypointensity in the middle of the chest, close to the right lung, as well as an enlarged spleen. Thus, the patient was discharged and received treatment for *H. pylori* infection and the ulcer (20 mg omeprazole twice daily, 1000 mg amoxicillin twice daily, 500 mg clarithromycin twice daily and 1000 mg bismuth citrate four times daily, for 8 weeks).

The patient was admitted to the hospital for a third time on June 1, 2011. Gastroscopical examination identified a probable ulcer, with gastric lymphoma, on the fundus of the stomach that was determined by biopsy to be a chronic superficial inflammation of the fundus. The patient was negative for *H. pylori* and the ulcer was in the same position as in the previous examination. A CT scan demonstrated that the wall of the antrum was thickened and nodules consistent with pulmonary emphysema were present in the middle of the right lung. The patient was discharged and once again received treatment for the ulcer (orally, with 20 mg omeprazole, twice-daily, for 8 weeks).

**Figure 3:**
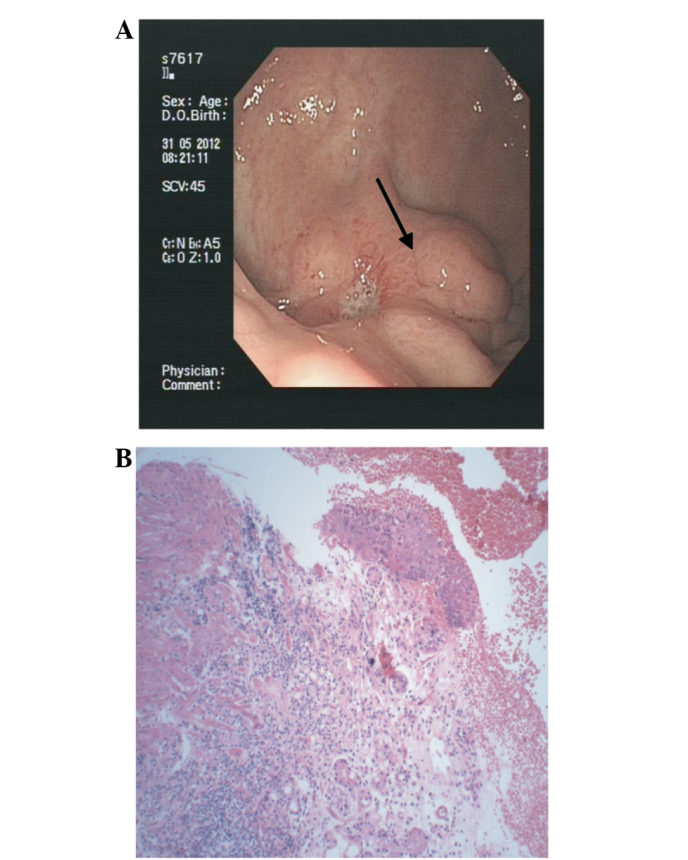
Case one: (A) Gastroduodenoscopy showing an ulcer on the fundus of the stomach (arrow) and (B) hematoxylin and eosin staining of the lesion revealing it to be an ulcer accompanied by inflammation (magnification, ×100).

The patient was admitted to the hospital for a fourth time on May 28, 2012. Gastroscopical examination indicated the presence of an ulcer, with gastric lymphoma, on the fundus of the stomach (Fig. 3A) that was confirmed by performing a biopsy (Fig. 3B). Furthermore, a CT scan demonstrated a thickened gastric wall and nodules in the middle of the right lung. The patient was again discharged and received additional anti-ulcer treatment (orally, with 20 mg omeprazole, twice-daily, for 8 weeks).

The patient was admitted to the hospital for a fifth time on April 7, 2013. Gastroscopical examination and biopsy identified an ulcer on the fundus of the stomach, with gastric lymphoma, and a CT scan demonstrated a thickened gastric wall that was similar to the previous examination and infection of the right lung. Once more, the patient was discharged and received anti-ulcer treatment (orally, with 20 mg omeprazole, twice-daily, for 8 weeks).

### Case two

**Figure 4:**
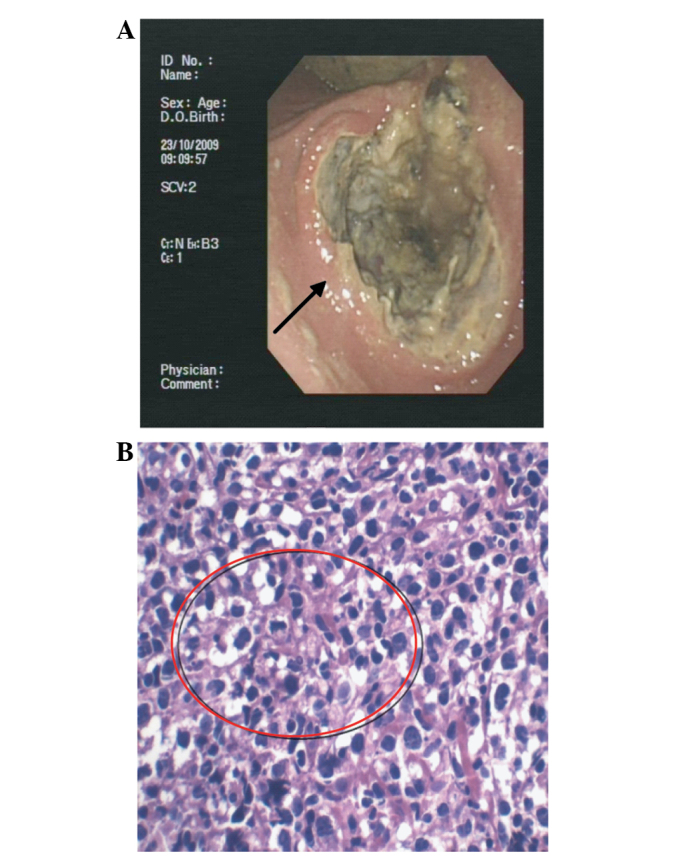
Case two: (A) Gastroduodenoscopy demonstrating a large ulcer of the antrum (arrow) and (B) hematoxylin and eosin staining of the lesion revealing a primary malignant gastric lymphoma confined to the mucosa (circled; magnification, ×400).

A 77-year-old female was admitted to the First Affiliated Hospital of Nanchang University on October 22, 2009, reporting a six-month history of upper abdominal distension, discomfort and intermittent diarrhea. Furthermore, the patient had a history of Parkinson’s disease, cholecystectomy, diabetes mellitus and hypertension. A CT scan demonstrated irregular thickening and stiffness in the wall of the antrum, as well as partial intrahepatic metastasis and bilateral pleural effusion with a small number of ascites. Gastroscopy (GIF-2T240; Olympus Corporation) indicated gastric cancer of the antrum (Fig. 4A), however, biopsy of the lesion determined it to be a distal gastric diffuse large B-cell lymphoma (Fig. 4B). The initial laboratory analyses revealed the following data (normal ranges are provided in parentheses): RBC, 2.39×10^12^/l (3.5–6×10^12^/l) and hemoglobin, 55 g/l (110–150 g/l). The patient received the following oral chemotherapeutic regimen: 375 mg/m^2^ rituximab and 5 mg dexamethasone on the first day; 0.5 mg VCR on the third and sixth days; 30 mg prednisone on the third and seventh days; and 750 mg/m^2^ CTX, 1.5 mg/m^2^ VCR and 50 mg/m^2^ Pharmorubicin on the tenth day.

The patient was admitted to the hospital for a second time on November 26, 2009, and received the following chemotherapeutic regimen: 375 mg/m^2^ rituximab on the first day; then a CHOP chemotherapy regimen consisting of 750 mg/m^2^ CTX, 1.5 mg/m^2^ VCR, 60 mg/m^2^ Pharmorubicin and 15 mg prednisone. CHOP chemotherapy was administered twice-daily from the second day onwards. Symptoms associated with the ulcer were relieved by regular anti-ulcer treatment prior to discharge. This included ondansetron to prevent vomitting, recombinant human granulocyte colony-stimulating factor to accelerate white blood cell production and omeprazole for anti-ulcer treament, until the symptoms subsided.

The patient was admitted to the hospital for a third time on December 24, 2009. Gastroscopical examination identified a lesion in the antrum and pyloric obstruction, while a biopsy of the lesion demonstrated inflammation. The patient received a third round of CHOP chemotherapy, followed by daily radiotherapy at 1.8 Gy for 22 days. Thus, radiotherapy was administered daily from February 8–17, 2010 and from March 8–19, 2010.

**Figure 5:**
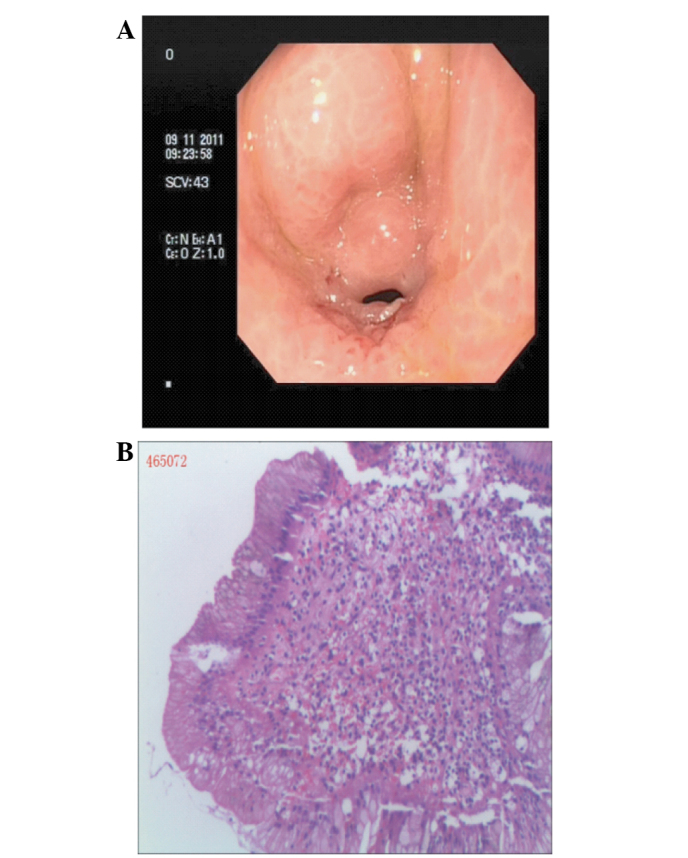
(A) Gastroduodenoscopy demonstrating an ulcer on the pyloric canal and (B) hematoxylin and eosin staining of the lesion revealing it to be an ulcer accompanied by inflammation (magnification, ×100).

The patient was admitted to the hospital for a fourth time on October 31, 2011, with a two-year history of repeated epigastric pains and a one-week history of nausea with vomiting. Gastroduodenoscopy identified an ulcer in the pyloric canal (Fig. 5A), which was confirmed by performing a biopsy (Fig. 5B). A CT scan demonstrated a thickened gastric wall and nodules in the middle of the right lung. The patient was discharged and received anti-ulcer treatment (orally, with 20 mg omeprazole, twice-daily, for 8 weeks).

**Figure 6:**
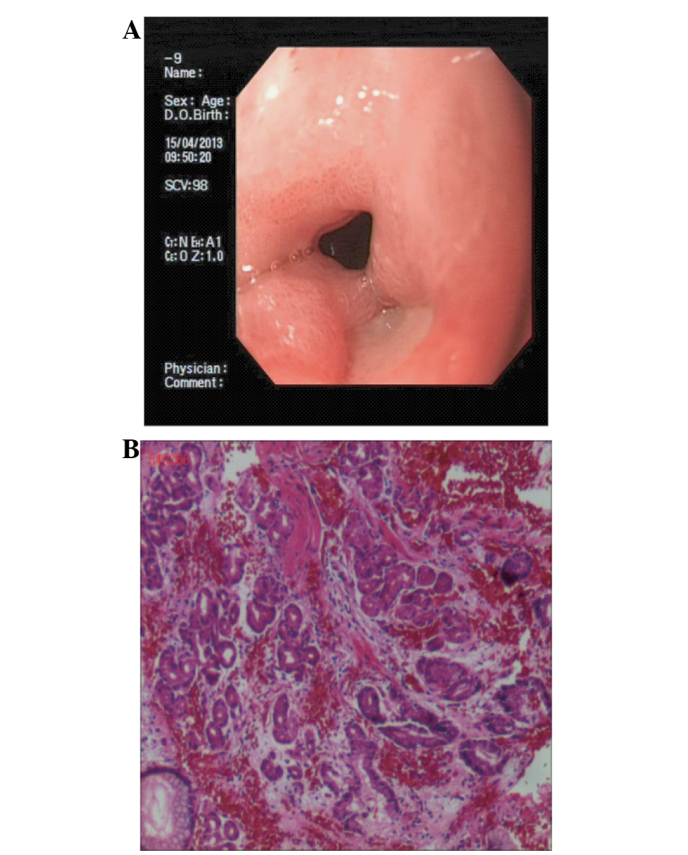
(A) Gastroduodenoscopy demonstrating an ulcer on the pyloric canal and (B) hematoxylin and eosin staining of the lesion revealing it to be an ulcer accompanied by inflammation of the pyloric canal (magnification, ×200).

Between September 2012 and April 2013, the patient was admitted back to the hospital three times for abdominal distension, nausea and vomiting. Gastroscopical examinations were performed each time and identified an ulcer at the same location (Fig. 6A), as determined by biopsy (Fig. 6B). The patient received treatment to alleviate the symptoms (ondansetron to prevent vomitting, recombinant human granulocyte colony-stimulating factor to accelerate white blood cell production and omeprazole for anti-ulcer treament, until the symptoms subsided), and was discharged each time.

## Discussion

It is difficult to determine the optimum primary treatment strategy for B-cell lymphoma of the mucosa-associated lymphoid tissue in the gastric marginal zone. This is partly due to the rarity of the disease, which only permits randomized clinical trials containing small numbers of patients to be performed. Furthermore, favorable outcomes resulting from various treatments (for example, surgery, chemotherapy, radiotherapy, antibiotic therapy and anti-CD20 immunotherapy) make comparisons complex (8,9).

In the present study, only the patient described in case one received a gastrectomy during the initial hospital stay, however, both of the patients received chemotherapy and radiotherapy. Although the sensitivity of gastric lymphomas to chemotherapy is well documented, no standard treatment exists (10–12). Radiotherapy and surgery have the advantage of being localized treatments, however, radiotherapy and chemotherapy are relatively low risk; for example, radiotherapy is administered over a relatively short duration and has minimal toxicity at the low doses currently used. However, there is great opposition to irradiating the stomach due to the risks of perforation and bleeding, renal toxicity and secondary malignancies (13). The risk of gastric bleeding and perforation during chemotherapy and/or radiotherapy in patients that do not undergo resection is 4%, whereas the risk is ~5% for patients undergoing gastrectomy (14). Bleeding and perforation predominantly occur in cases of advanced disease, and the risk is comparable to the perioperative risk of mortality associated with the gastrectomy procedure itself (15–17).

The two patients in the present study developed refractory *H. pylori*-negative peptic ulcers following radiotherapy. Despite regular anti-ulcer therapy and symptomatic treatment, the size and position of the ulcers remained unchanged. Refractory peptic ulcers appear to stem from complications of radiotherapy for malignant gastric lymphomas. Thus, novel and effective therapies should be explored to treat peptic ulcers following radiotherapy in this patient population, as symptomatic treatment is currently the only option.

## References

[1] Dragosics B, Bauer P, Radaszkiewicz T (1985). Primary gastrointestinal non-Hodgkin’s lymphomas. A retrospective clinicopathologic study of 150 cases. Cancer.

[2] Radaszkiewicz T, Dragosics B, Bauer P (1992). Gastrointestinal malignant lymphomas of the mucosa-associated lymphoid tissue: factors relevant to prognosis. Gastroenterology.

[3] Paryani S, Hoppe RT, Burke JS, Sneed P, Dawley D, Cox RS, Rosenberg SA, Kaplan HS (1983). Extralymphatic involvement in diffuse non-Hodgkin’s lymphoma. J Clin Oncol.

[4] Aisenberg AC (1995). Coherent view of non-Hodgkin’s lymphoma. J Clin Oncol.

[5] Severson RK, Davis S (1990). Increasing incidence of primary gastric lymphoma. Cancer.

[6] Koch P, Probst A, Berdel WE, Willich NA, Reinartz G, Brockmann J, Liersch R, del Valle F, Clasen H, Hirt C (2005). Treatment results in localized primary gastric lymphoma: data of patients registered within the German multicenter study (GIT NHL 02/96). J Clin Oncol.

[7] Ferrucci PF, Zucca E (2007). Primary gastric lymphoma pathogenesis and treatment: what has changed over the past 10 years?. Br J Haematol.

[8] Swerdlow SH, Campo E, Seto M, Swerdlow SH, World Health Organization (2008). Mantle cell lymphoma. WHO Classification of Tumours of Haematopoietic and Lymphoid Tissues.

[9] Nakamura S, Matsumoto T, Iida M, Yao T, Tsuneyoshi M (2003). Primary gastrointestinal lymphoma in Japan: a clinicopathologic analysis of 455 patients with special reference to its time trends. Cancer.

[10] Fischbach W, Schramm S, Goebeler E (2011). Outcome and quality of life favour a conservative treatment of patients with primary gastric lymphoma. Z Gastroenterol.

[11] Ouakaa-Kchaou A, Gargouri D, Elloumi H, Kochlef A, Bouzid H, Kilani A, Romani M, Kharrat J, Ghorbel A (2011). Survival in patients with gastric lymphoma. Tunis Med.

[12] Yoshita H, Sugiyama T (2012). Gastric malignant lymphoma. Nihon Rinsho.

[13] Nam TK, Ahn JS, Choi YD, Jeong JU, Kim YH, Yoon MS, Song JY, Ahn SJ, Chung WK (2014). The role of radiotherapy in the treatment of gastric mucosa-associated lymphoid tissue lymphoma. Cancer Res Treat.

[14] Liu HT, Hsu C, Chen CL, Chiang IP, Chen LT, Chen YC, Cheng AL (2000). Chemotherapy alone versus surgery followed by chemotherapy for stage I/IIE large-cell lymphoma of the stomach. Am J Hematol.

[15] Selçukbiricik F, Tural D, Elicin O, Berk S, Ozgüroğlu M, Bese N, Ferhanoglu B (2012). Primary gastric lymphoma: conservative treatment modality is not inferior to surgery for early-stage disease. ISRN Oncol.

[16] García M, Bellosillo B, Sánchez-González B, García-Payarols F, Seoane A, Ferrer AM, Gimeno E, Barranco LE, Torner A, Solé F (2012). Study of regulatory T-cells in patients with gastric malt lymphoma: influence on treatment response and outcome. PLoS One.

[17] Shimada S, Gen T, Okamoto H (2013). Malignant gastric lymphoma with spontaneous perforation. BMJ Case Rep.

